# Bacteroides thetaiotaomicron With Concomitant Streptococcus milleri as a Cause of Purulent Pericarditis: A Case Report

**DOI:** 10.7759/cureus.63153

**Published:** 2024-06-25

**Authors:** Fawaz Mohammed, Sajjad Haider, Muhammad Akbar, Sameer Saleem, Charles Lin

**Affiliations:** 1 Internal Medicine, University of Kentucky College of Medicine, Bowling Green, USA; 2 Cardiology, University of Kentucky College of Medicine, Bowling Green, USA; 3 Internal Medicine, Medical Center at Bowling Green, Bowling Green, USA; 4 Cardiology, Medical Center at Bowling Green, Bowling Green, USA

**Keywords:** impending cardiac tamponade, streptococcus milleri, bacteroides species, recurrent pericarditis, purulent pericarditis

## Abstract

Purulent pericarditis secondary to bacterial infections is rarely seen in the current era of broad-spectrum antibiotics. Complications tend to be higher in comparison to viral or idiopathic etiologies in cases of bacterial pericarditis. *Staphylococcal aureus, Streptococcus pneumoniae, *or *Haemophilus influenza *are the more commonly identified pathogens in cases of bacterial pericarditis. We report a case of purulent pericarditis from *Bacteroides thetaiotaomicron *and *Streptococcus milleri *occurring in conjunction in a 56-year-old male. To our knowledge, there are no published case reports describing purulent pericarditis from *B. thetaiotaomicron *and *S. milleri *occurring simultaneously in the literature.

## Introduction

Pericarditis, when occurring from bacterial causes, tends to have poorer outcomes compared to other causes of pericarditis [1]. Infection of the pericardium can occur from hematogenous spread or contiguous spread from nearby surrounding structures in the thoracic cavity. Staphylococcus aureus is the most common causative organism. Other common causes of bacterial pericarditis (BP) are *Streptococcus pneumoniae, Haemophilus influenza, *and* Neisseria meningitides* [2]. Identifying the causative organism early is important to allow for better outcomes. We report the case of a 56-year-old man who was found to have a large pericardial effusion with pericardial fluid cultures yielding *Bacteroides thetaiotaomicron *and *Streptococcus milleri* species occurring in conjunction.

## Case presentation

**Figure 1:**
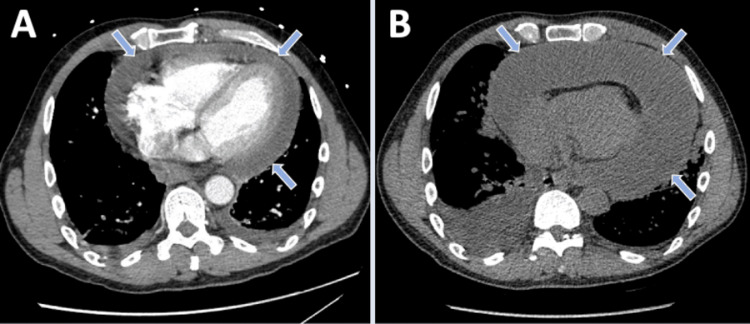
Chest computed tomography findings (A) Chest CT from index admission showing moderate-sized pericardial effusion (arrows). (B) Chest CT from the second admission with large pericardial effusion increased in size from the previous (arrows).

**Figure 2:**
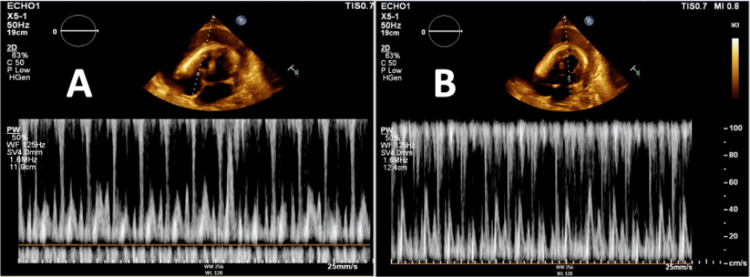
Transthoracic echocardiogram showing tricuspid and mitral valve inflow patterns (A) Respiratory variability in tricuspid inflow velocity less than 40%. (B) Respiratory variability in mitral valve inflow velocity greater than 25% consistent with impending tamponade.

A 56-year-old male with a recent medical history significant for viral pericarditis secondary to rhinovirus infection who was discharged with indomethacin and colchicine for viral pericarditis two weeks back presented for a follow-up appointment with his cardiologist in the outpatient setting and complained of worsening shortness of breath and bilateral lower extremity edema. With his worsening symptoms in the setting of previous pericarditis with pericardial effusion, he was instructed to go to the hospital for further management. He denied symptoms of chest pain, palpitations, lightheadedness, fevers or chills. On presentation, he had a blood pressure of 94/63 mmHg, a heart rate of 89/min, and a respiratory rate of 20/min saturating appropriately on room air. A chest computed tomography (CT) was performed which showed a large pericardial effusion, increased in size from previous pericardial effusion (Figures 1A, 1B). Cardiology was consulted and the patient underwent emergent pericardiocentesis given findings of impending tamponade on transthoracic echocardiogram (TTE) with drainage of one liter of cloudy yellow-green fluid (Figures 2A, 2B).

**Figure 3:**
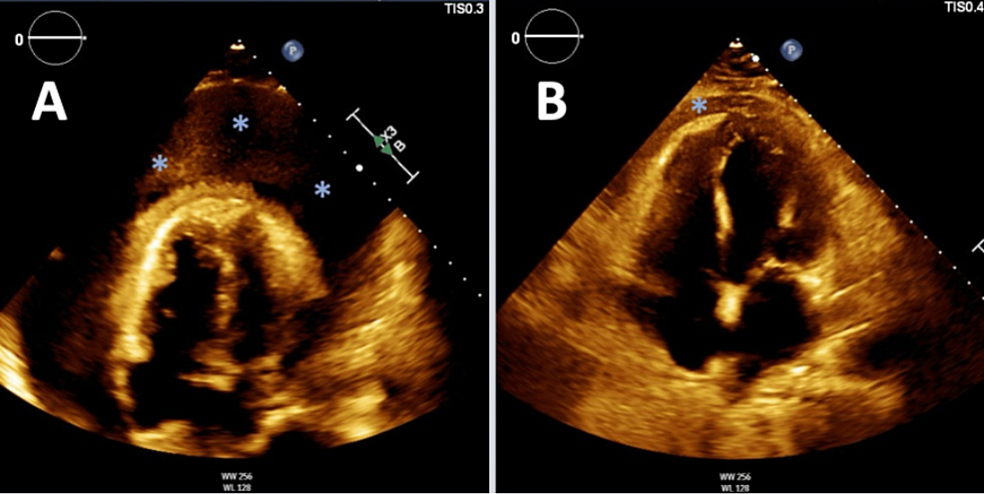
Transthoracic echocardiogram apical four-chamber view (A) TTE on presentation showing large pericardial effusion with impending tamponade (asterixis). (B) TTE following pericardiocentesis showing trace pericardial effusion (asterixis).

TTE after pericardiocentesis showed trace pericardial effusion (Figures 3A, 3B). Cell count was 171,000 (0-5/µL) consistent with purulent pericarditis. Pericardial fluid cultures yielded *B. thetaiotaomicron* and *S. milleri*. Infectious disease was consulted the patient was initiated on appropriate antibiotics. There was concern for possible seeding from a fistulous connection between the pericardium and gastrointestinal tract as the cause of pericarditis. An esophagogram was obtained which showed no evidence of leak into the mediastinum. Cardiothoracic surgery was consulted and given the patient had persistent purulent pericarditis with the development of constrictive pericarditis, the decision was made to perform pericardiectomy. A pericardiectomy was performed and he received six weeks of intravenous antibiotics. His symptoms improved and after completion of the antibiotic regimen, a follow-up echocardiogram was performed which showed resolution of the pericardial effusion.

## Discussion

Purulent pericarditis occurs commonly as a secondary infection from direct spread from surrounding structures into the thoracic cavity, the most common being a lung source [3]. It is characterized by the presence of frank pus in the pericardium. Solitary infection of the pericardium has been rarely reported. Conditions that may predispose individuals to infection of the pericardium are underlying malignancy, recent thoracic surgery, or an antecedent pericardial effusion as seen in our case [4]. If not identified and treated promptly the prognosis is grim with a reported mortality rate of close to 100% [5]. Most cases occur due to gram-positive species namely *S. aureus*. Recent reports have suggested an increasing incidence of gram-negative bacteria as a cause [6]. *B. thetaiotaomicron* is an anaerobic bacterium that is part of the normal intestinal flora however their spread to sterile locations can lead to infection as seen in our case [7]. Factors implicated in infection from *B.* *thetaiotaomicron* include enterotoxin production and the presence of lipopolysaccharide in the cell envelope. On the other hand, S. milleri is a facultative anaerobic bacterium that is confined within the oral cavity and is rarely known to cause significant pathology [8]. Several virulence factors pertaining to the bacteria, including fibronectin-binding protein and hyaluronidase, have been implicated in human infection. Recognizing BP promptly can be difficult as classic findings like pericardial friction rub and pulsus paradoxus are not always present and the clinical presentation is often with nonspecific signs and symptoms. Although cardiac tamponade can be recognized with constellation of clinical findings, TTE is of great utility [9]. Not only does it assist in diagnosis but can help with prompt removal of the pericardial fluid which correlates with better rates of survival [9]. Pericardiocentesis is followed by tailored antibiotic therapy. Immediate pericardiocentesis and antibiotic therapy are adequate in most cases, however, the presence of thick loculated fluid can complicate treatment. In these cases, the creation of a pericardial window with irrigation or pericardiotomy may be needed [1]. If treatment is delayed, constrictive pericarditis may develop. In such instances, pericardiectomy is performed. Intrapericardial fibrinolysis has been proposed as a modality to prevent persistent purulent pericarditis and constrictive pericarditis given the morbidity associated with pericardiectomy [10]. To our knowledge, our case is the first case of purulent pericarditis from *B. thetaiotaomicron *and *S. milleri *occurring in conjunction. It was likely that the patient had seeding of these bacteria into the mediastinal space from a transient breach in the mucosal tissue of the esophageal cavity.

## Conclusions

Purulent pericarditis, a rare form of pericarditis carries a high risk of morbidity and mortality. Prompt recognition and treatment are warranted given the poor prognosis with delayed treatment. Infection from aerobic bacteria is typically seen; however, rarely, anaerobic bacteria can be isolated, as seen in our case, warranting suspicion for an intra-abdominal source of infection. Tailored antibiotics are the cornerstone of treatment with surgical intervention warranted in cases of persistent or recurrent pericarditis.
